# Soft Tissue Repair with Easy-Accessible Autologous Newborn Placenta or Umbilical Cord Blood in Severe Malformations: A Primary Evaluation

**DOI:** 10.1155/2017/1626741

**Published:** 2017-12-17

**Authors:** Åsa Ekblad, Magdalena Fossum, Cecilia Götherström

**Affiliations:** ^1^Department of Clinical Science, Intervention and Technology, Division of Obstetrics and Gynecology, Karolinska Institutet, Stockholm, Sweden; ^2^Department of Women's and Children's Health at Centre of Molecular Medicine, Karolinska Institutet, Stockholm, Sweden; ^3^Patient Area Children with Diseases of the Abdomen and Blood or Cancer, Section of Urology, Astrid Lindgren Children's Hospital, Karolinska University Hospital, Stockholm, Sweden

## Abstract

Disrupted organogenesis leads to permanent malformations that may require surgical correction. Autologous tissue grafts may be needed in severe lack of orthotopic tissue but include donor site morbidity. The placenta is commonly discarded after birth and has a therapeutic potential. The aim of this study was to determine if the amnion from placenta or plasma rich of growth factors (PRGF) with mononuclear cells (MNC) from umbilical cord blood (UCB), collected noninvasively, could be used as bio-constructs for autologous transplantation as an easy-accessible no cell culture-required method. Human amnion and PRGF gel were isolated and kept in culture for up to 21 days with or without small intestine submucosa (SIS). The cells in the constructs showed a robust phenotype without induced increased proliferation (Ki67) or apoptosis (caspase 3), but the constructs showed decreased integrity of the amnion-epithelial layer at the end of culture. Amnion-residing cells in the SIS constructs expressed CD73 or pan-cytokeratin, and cells in the PRGF-SIS constructs expressed CD45 and CD34. This study shows that amnion and UCB are potential sources for production of autologous grafts in the correction of congenital soft tissue defects. The constructs can be made promptly after birth with minimal handling or cell expansion needed.

## 1. Introduction

Organogenesis occurs early during embryonic development, and disruption of normal organogenesis results in permanent congenital malformations [1]. These malformations affect up to 3% of all infants [2] and are one of the leading causes of neonatal death [3]. Congenital malformations are also associated with morbidity and are responsible for a significant part of pediatric hospital admissions and costs [4]. Correction of congenital soft tissue defects such as myelomeningocele (MMC), giant omphalocele, gastroschisis, and congenital diaphragmatic hernia (CDH) involves surgical correction short after birth. Affected newborns without spare tissue are dependent of donor tissue. Because of the worldwide perinatal organ donor shortage [1], prosthetic materials are sometimes being used [5, 6], which might be associated with complications such as recurrence, graft dislodgement and/or rejections, erosion of the graft, and infections [7–9]. Biological graft material would be preferable and consists of the extracellular compartment of human or animal tissue. They are also associated with a risk for recurrence but have the advantage of tissue remodeling, neovascularization, and lower rate of infections [10, 11] and also advantages in the situation of high-risk contamination areas, such as congenital high-risk abdominal wall defects [12].

The progress in tissue engineering (TE) paves an alternative route to the traditional use of prosthetic materials [13]. The most common approach involves a combination of biological matrices and cells. Populating the matrix with cells enhances graft integration and restoration of host tissue and reduces scar formation and bacterial infections [13, 14]. Small intestine submucosa (SIS) consists of cell-free porcine extracellular matrix (ECM) (mostly collagens), an absorbable biomaterial that contains growth factors and supports angiogenesis, frequently used for surgical correction of soft tissue defects [15, 16].

Autologous grafts are considered as the gold standard, but they often include donor site morbidity where healthy tissue is removed from one site of the body to replace injured or missing tissue [17] and have limitations depending on the graft area size. Allogeneic grafts eliminate the donor site morbidity but may induce an immune response in the recipient [18], rejection with subsequent necrosis [19], and allograft removal or replacement.

The placenta is a fetomaternal organ consisting of three layers: outer maternal decidua, fetal chorion membrane and inner fetal amnion membrane surrounding the fetus, and the amniotic fluid. The human placenta has been used in medicine since the 1500s for the treatment of, for example, leg ulcers, wounds, and in skin transplantation [20–24]. The amnion contains multipotent cells, both in the epithelial and the mesenchymal layers, and possesses anti-inflammatory and regenerative properties and also antifibrotic, antimicrobial, immune suppressive, and angiogenic properties [25–29]. Umbilical cord blood (UCB) contains hematopoietic stem cells and has been used for transplantation to cure malignant and other severe diseases for more than 30 years [30]. From UCB, plasma rich of growth factors (PRGF) can be isolated [31]. Both experimental and clinical studies have shown that PRGF is clinically safe and promotes vascularization, tissue regeneration, and wound healing [32, 33].

To overcome the problems with donor site morbidity, allogenic immunological reactions, and long production time, we hypothesized that autologous proangiogenic constructs could be produced from derivatives of the term placenta; amnion and PRGF gel with mononuclear cells (MNC) from UCB. Both are noninvasive sources for autologous tissue engineering that are easily available after birth with no ethical obstacles of their use.

The objective of this study was to analyze amnion and MNC-enriched PRGF gel with respect to tissue and cell integrity, morphology, and viability. In addition, amnion and PRGF gel were combined with SIS in order to improve mechanical strength and to build a durable graft. The tissue and cell integrity of the amnion and PRGF-SIS constructs were analyzed as above, but additionally for changes in cell morphology and protein expression.

## 2. Material and Methods

### 2.1. Isolation of the Amnion

Human term placentas (*n* = 4) were obtained after elective caesarian sections from healthy donors after informed oral and written consent according to ethical approval from the Regional Ethical Review Board in Stockholm (Dnr 2012/480-31/1) and in accordance with the Declaration of Helsinki. The placentas were placed in a sterile environment with the cord side up and transported to the laboratory. The amnion was mechanically peeled off from the chorion membrane starting from the cord. Amnion pieces were transferred to petri dishes and washed three times in phosphate buffered saline (PBS) (Life Technologies, Paisley, United Kingdom) before being further processed as described below.

### 2.2. Comparison of Culture Conditions of Amnion

To evaluate culture conditions, three different culture media were initially compared in regard to amnion-residing cell morphology, viability, and proliferation. The culture media compared were (1) mesenchymal stromal cell culture medium: modified Eagle medium alpha (MEM*α*) (Invitrogen, Carlsbad, CA, USA) supplemented with 10% inactivated fetal calf serum (FCS) (PAA Laboratories GmbH, Pasching, Austria); (2) epithelial cell culture medium (DMEM-EC): Dulbecco's modified Eagle's medium-high glucose (Invitrogen) supplemented with 10% FCS (PAA Laboratories), 2 mM L-glutamine (Sigma-Aldrich, St. Louis, MO, USA), 1% nonessential amino acid (Sigma-Aldrich), 55 *μ*M 2-mercaptoethanol (Sigma-Aldrich), and 1 mM sodium pyruvate (Sigma-Aldrich); and (3) endothelial cell culture medium (DMEM-F12): Dulbecco's Modified Eagle Medium/Nutrient Mixture F-12 (Invitrogen) supplemented with 10% FCS (PAA Laboratories) and 1% L-glutamine (Sigma-Aldrich). All culture media were supplemented with 1% antibiotic-antimycotic solution (Anti-Anti, Life Technologies). Approximately, 3 × 2 cm^2^ pieces of amnion were isolated and placed in 12-well cell culture plates (Becton & Dickinson, Franklin Lakes, NJ, USA) equally divided between the different culture conditions. The amnion pieces were maintained at 37°C in a humidified environment containing 5% CO_2_, and the media was replaced every 3-4 days. At days 0, 3, 7, 10, 14, and 21, the amnion pieces were washed twice in PBS and fixed in 4% paraformaldehyde solution (PFA) (Solveco AB, Rosersberg, Sweden) for 3 days. Thereafter, the amnion pieces from all time points and culture conditions were paraffin embedded, sectioned, and stained with hematoxylin (HTX) (Meyers HTX, HistoLab Products AB, Gothenburg, Sweden) and eosin for morphology (0.2%, HistoLabs Products AB), Ki67 for proliferation, (1 : 500, Life Technologies), and activated caspase 3 for apoptosis (1 : 200, Invitrogen). To visualize the staining, biotinylated secondary anti-mouse IgG antibodies were used (1 : 500, Vector Laboratories Inc., Burlingame, CA, USA). Avidin-Biotin complex (Vector Laboratories Inc.) was added and subsequently DAB (Dako, Glostrup, Denmark). The sections were counterstained with HTX (HistoLab Products AB).

### 2.3. Characterization of Migrated Amnion-Derived Cells in the Different Culture Conditions

To characterize amnion-derived cells (ADC) that migrated out from the tissue and adhered to the cell culture surface, the ADC was analyzed with flow cytometry at day 21. The cells were harvested by treatment with 0.05% trypsin and 0.53 mM EDTA (Invitrogen) and resuspended in PBS. The ADC were incubated with conjugated monoclonal antibodies against CD31, CD73 (Becton-Dickinson), and PCK (Dako) for 30 min in the dark at 4°C. The antibodies were conjugated to fluorescein (FITC), phycoerythrin (PE), or cyanine 3 (Cy3). Thereafter, the cells were washed and resuspended in PBS. The cells were analyzed with a flow cytometer with 488 nm excitation and emission filters (FACSCalibur, Becton-Dickinson), and the data was analyzed using the software Flow-Jo (v. 10, Tree Star Inc., Ashland, OR, USA).

### 2.4. Characterization of Amnion-Residing Cells in Constructs with SIS

To analyze the properties of the amnion-residing cells in the construct with SIS (amnion-SIS), 2 × 2 cm^2^ pieces of amnion were placed on dry 4-layer SIS (Biodesign® Tissue Graft, Cook Biotech Incorporated, IN, USA) and let adhered for 5 minutes. To increase the contact, the amnion was sutured onto the SIS matrix (Prolene 6-0, Ethicon Inc., NJ, USA) (Table 1), and the construct was kept in culture for up to 21 days in DMEM-EC medium. At days 0, 3, 7, 10, 14, and 21 amnion-SIS pieces were washed twice and transferred to 4% PFA (Solveco AB) for fixation and thereafter paraffin embedded and sectioned. The sections were stained with HTX/eosin (HistoLab Products AB) for morphology or pan-cytokeratin (PCK, epithelium 1 : 500, Dako), CD31 (endothel, 1 : 500, Dako), and CD73 (common stromal marker, 1 : 500, Abcam, Cambridge, United Kingdom) for surface epitope expression, and Ki67 (1 : 500, Life Technologies) and activated caspase 3 (1 : 200, Invitrogen) as above. Stained sections of amnion without SIS cultured in DMEM-EC served as a control.

### 2.5. Preparation of Cell-Enriched Gel from Plasma Rich in Growth Factors

Term UCB was collected after birth, before delivery of the placenta, by puncturing the umbilical vein using a sterile collection system (Macopharma MSC1206DU cord blood collection kit, Tourcoing, France) containing 21 mL anticoagulant (citrate phosphate dextrose solution). The UCB was collected by gravity until flow ceased. Fresh unbanked (because of too low cell counts) UCB units (*n* = 3, mean total volume 109 ± 15 mL) were obtained from the Swedish National Cord Blood bank, Karolinska University Hospital, Sweden, after informed oral and written consent according to ethical approval from the Regional Ethical Review Board in Stockholm (Dnr 2012/480–31/1) and in accordance with the Declaration of Helsinki.

To produce a gel from PRGF, 45 mL of the UCB was centrifuged at 500*g* for 10 min at room temperature. The UCB separated into red blood cells at the bottom of the tube, PRGF in the middle layer, and plasma poor in growth factors (PPGF) in the top layer. 10 mL of the PPGF layer was discarded, and the remaining PRGF, including 3-4 mm of the RBC fraction to maximize the platelet recovery [34], was transferred to a new tube. To enrich the PRGF with a high concentration of cells, the remaining UCB was diluted four times with PBS and the MNC were isolated by density gradient separation (Lymphoprep, density 1.077 g/mL, Axis-Shield PoC AS, Oslo, Norway). The MNC were counted in Türk's solution (Merck, KGaA, Darmstadt, Germany) and resuspended to 60 × 10^6^ cells/mL in PRGF. 42 *μ*L of 10% CaCl_2_ in distilled water per mL PRGF was added for gel formation.

### 2.6. Characterization of PRGF-Residing Cells in Constructs with SIS

To evaluate the durability of the produced gel and to analyze the protein expression of the MNC in the PRGF, pieces of SIS (0.9 cm^2^) were placed in 8-chamber slides (Merck Millipore, Cork, Ireland) and 350 *μ*L PRGF was added on top and incubated for 30–45 min in room temperature until gel was formed (Table 1). PRGF (1 mL) without SIS was produced as a control. The gel pieces and the PRGF-SIS constructs were transferred to 12-well plates (Becton-Dickinson) and 350 *μ*L RPMI 1640 medium (Life Technologies) supplemented with 10% pooled inactivated human AB serum, 100 U/mL penicillin, and 100 mg/mL streptomycin (HyClone Laboratories, South Logan, UT, USA), and 20 mM L-glutamine (Invitrogen) was added per well. At days 0, 3, 7, 10, and 14, the PRGF gel and PRGF-SIS constructs were collected, washed in PBS, and transferred to 4% PFA for 3 days and thereafter paraffin embedded and sectioned. The sections were stained as above with HTX/eosin for morphology, CD34 and CD45 (both hematopoietic surface markers, 1 : 100, Dako) for surface epitope expression, Ki67 (1 : 500, Life Technologies), and for activated caspase 3 (1 : 250, Invitrogen).

### 2.7. Statistical Analysis

Data from the flow cytometry analysis were analyzed using one factor analysis of variance (ANOVA) and paired Student *t*-test, assuming unequal variances between samples. Results were considered statistically significant when *p* values were <0.05.

## 3. Results

### 3.1. Isolation and Culture of the Amnion

**Figure 1:**
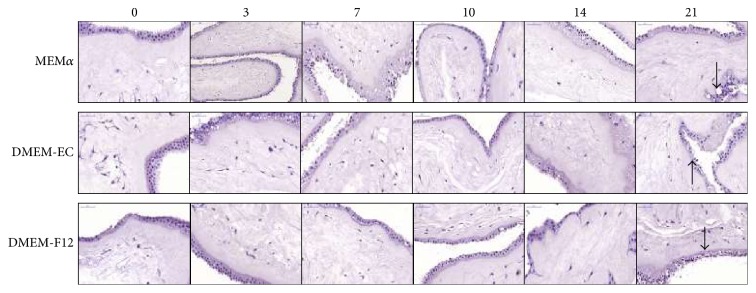
Morphology of amnion-residing cells. Pieces of amnion were kept in culture for up to 21 days in three different culture media and thereafter stained with HTX/eosin. The different culture conditions did not induce morphological changes, but there was a decreased integrity of the epithelial layer integrity over time (indicated by the arrows). Magnification 20x.

**Figure 2:**
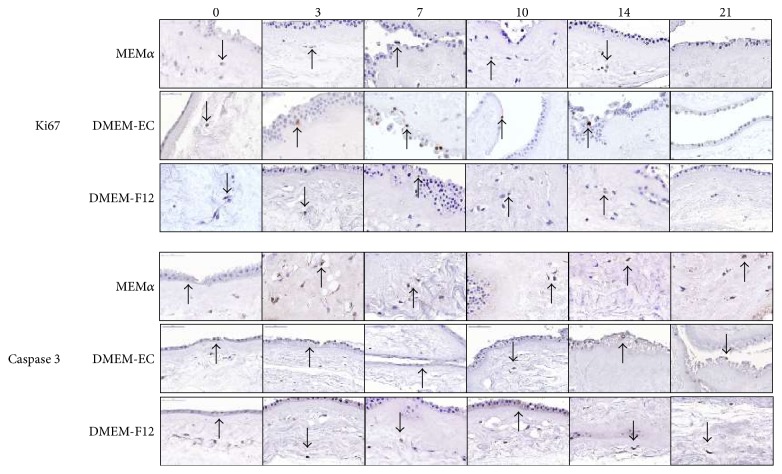
Proliferation and viability of amnion-residing cells *in vitro*. Pieces of amnion were kept in culture for up to 21 days in three different culture media and thereafter stained for Ki67 (proliferation) and caspase 3 (apoptosis) at days 0 to 21. There was no significant difference between the culture conditions. A low expression of Ki67 and caspase 3 was detected up to day 14, and on day 21, Ki67 was not detected and caspase 3 showed a slight increased expression. Single positive cells stain brown as indicated by the arrows. Magnification 60x.

**Figure 3:**
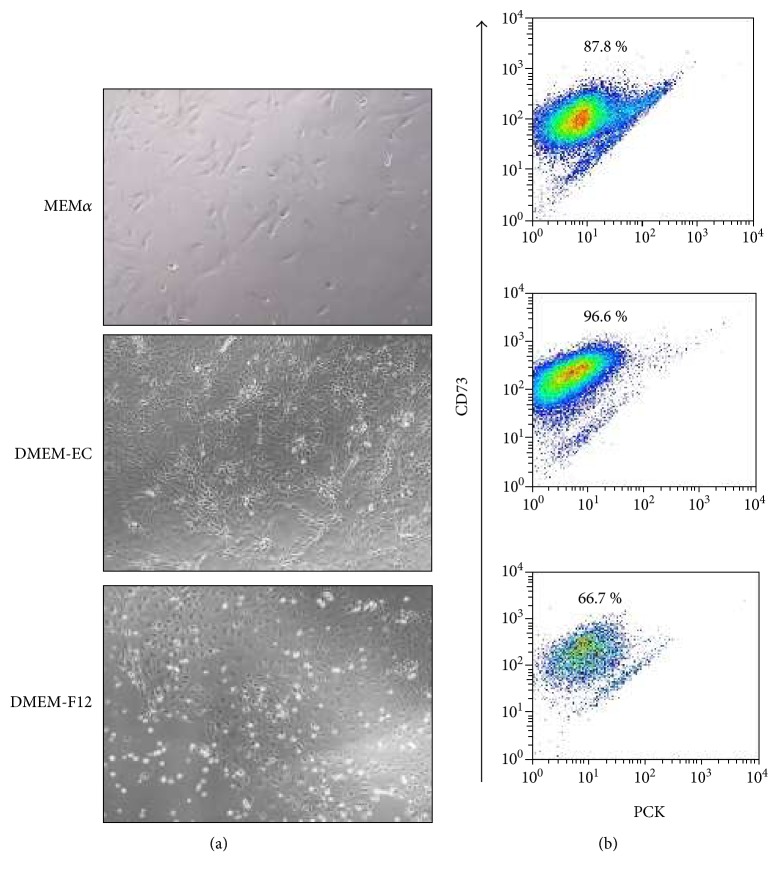
Properties of amnion-derived cells (ADC). ADC migrated from cultured amnion and adhered onto the plastic culture surface. (a) Typical images of the morphology of the ADC at day 14 in the different culture conditions. Magnification 10x. (b) Cell surface expression of CD73 and PCK by ADC was analyzed by flow cytometry. Typical plots are shown with the mean CD73 expression in percent.

The amnion could easily be isolated from the placentas and kept in culture to the endpoint of 21 days. To evaluate different culture media, amnion pieces were cultured in MEM*α*, DMEM-EC, and DMEM-F12. The amnion pieces were evaluated morphologically with HTX/eosin, which showed that the mesenchymal cells had maintained morphology throughout the time in culture, but there was a decreasing integrity of the epithelial layers at the end of culture in all conditions (Figure 1). There were no differences in proliferation and viability of the amnion-residing cells when testing the different culture media. A uniform low expression of Ki67 was detected at all time points up to day 21 when no expression was detected (Figure 2). Expression of caspase 3 was low, but slightly increased over time (Figure 2). The ADC in MEM*α* and DMEM-EC showed fibroblast-like morphology whereas the cells in DMEM-F12 had neither typical epithelial-like morphology nor fibroblast-like morphology (Figure 3(a)). The ADC were further investigated by surface epitope expression by flow cytometry, and the majority of cells from the different culture conditions expressed CD73 with a significantly higher expression in DMEM-EC compared to the other cell culture media (DMEM-F12 66.7%, MEM*α* 87.8%, and DMEM-EC 96.6%, *p* = 0.03 and 0.008, resp.) (Figure 3(b)). There was no expression of CD31 or PCK of the ADC in any of the culture conditions (data not shown). Based on these results, DMEM-EC was selected for the remaining experiments.

### 3.2. Properties of Amnion-SIS Constructs

**Figure 4:**
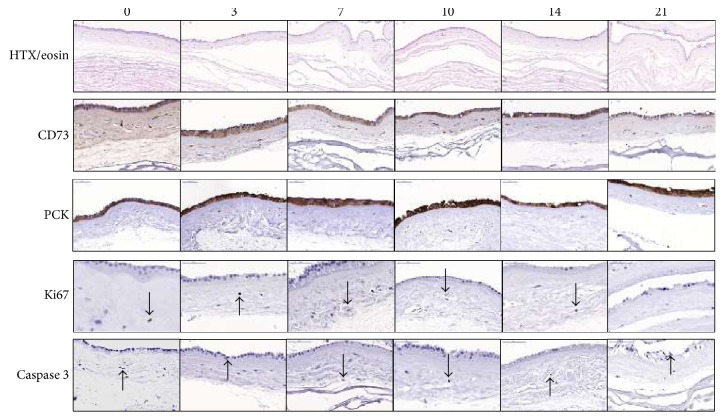
Morphology and protein expression in amnion-SIS constructs. Amnion sutured to SIS was analyzed for protein expression at days 0 to 21. All amnion-residing cells expressed CD73, and epithelial cells expressed PCK at all time points (brown staining). A low expression of Ki67 and caspase 3 was detected up to day 14. On day 21, Ki67 was not detected and caspase 3 showed a slight increased expression. Single positive cells stain brown as indicated by the arrows. Magnification 40x (CD73 and PCK) and 60x (Ki67 and caspase 3).

To investigate whether the amnion-residing cells would migrate and integrate into SIS and to analyze protein expression, amnion pieces were combined with SIS and kept in culture for 0–21 days and thereafter stained for protein expression. Amnion without SIS served as a control. The HTX/eosin staining showed no morphological differences compared to amnion pieces without SIS, and no cells were detected in the SIS. Cells in both the epithelial and mesenchymal layers expressed CD73, and the epithelial cell layer expressed PCK. There was a low expression of Ki67; less than 3% of the cells at all time points. At day 0, a few cells within the inner mesenchymal layer expressed Ki67, and at later time points, no Ki67-positive mesenchymal cells were detected. At day 0, the epithelial layer contained no Ki67-positive cells, but the Ki67 expression increased with a peak at day 7. At day 21, there were no Ki67-positive cells detected in any samples. Overall, there was a low expression of caspase 3 in both mesenchymal and epithelial cells, but it slightly increased in the epithelial cells over time. There was no CD31 expression at any time points (data not shown). There were no differences in protein expression between amnion-SIS and amnion without SIS (Figure 4 and Supplementary Figure 1).

### 3.3. Properties of PRGF-SIS Constructs

**Figure 5:**
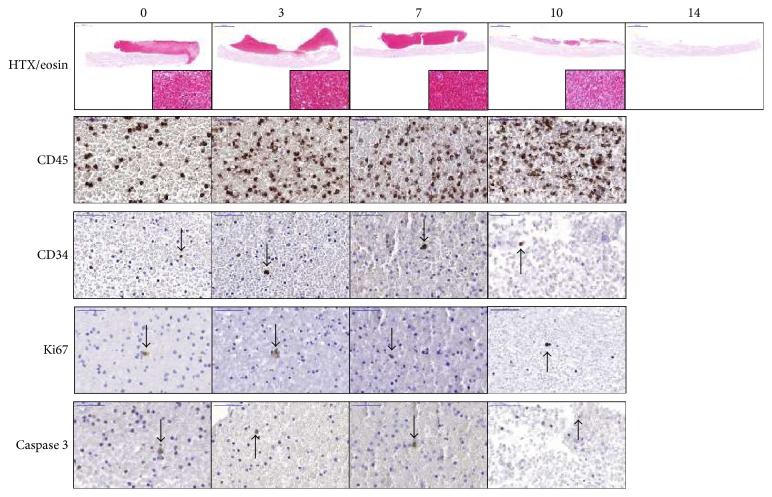
Morphology and protein expression in PRGF-SIS constructs. The PRGF-SIS constructs were kept in culture for up to 14 days and thereafter analyzed. The HTX/eosin staining showed decreasing number of cells and a complete degradation of the gel at day 14. The cells expressed CD45 (brown staining), and few CD34-positive cells were detected throughout the time points. Ki67-positive cells were detected, but with decreasing number over time. The caspase 3 expression was low but increased over time. Single positive cells stain brown as indicated by the arrows. Magnification 2x and 60x.

The PRGF gel with or without SIS could be kept in culture for up to 14 days with sustained morphology, but with decreasing number of cells and with an increasing degradation of the gel. At day 14, the gel was completely dissolved and only SIS remained. Therefore, day 14 was excluded from further analysis. There were no differences in protein expression if the PRGF gel was combined with SIS or not. The cells expressed CD45, and a few CD34-positive cells could be detected at all time points. Cells were positive for Ki67, but with decreasing number over time. The caspase 3 expression was low but increased over time (Figure 5 and Supplementary Figure 2).

## 4. Discussion

Conventional harvesting of autologous biological starting material for TE involves at least one invasive donor site, leading to at least two sites of surgical interventions. To circumvent the donor site morbidity, a noninvasive technique for collection is desirable. In this study, we investigated two highly proangiogenic types of autologous constructs made from the noninvasive source term placenta: (1) amnion and (2) MNC-enriched PRGF gel from UCB.

This is, to our knowledge, the first study on fresh amnion and PRGF combined with a biological graft. Our results demonstrate that the placenta and UCB can easily be collected directly after birth and the amnion can be separated from the chorion and is ready for use in less than one hour after birth. The PRGF can be produced within 30 min after retrieving the UCB, isolation of MNC took up to one hour with minimal addition of compliant products (Lymphoprep and CaCl_2_), and gel formation occurred within 45 min. In total, a complete MNC-enriched PRGF construct can be prepared within 2-3 hours after birth. At this point, the constructs can be used for surgical correction of the defect.

We wanted to investigate if the properties of the amnion were maintained after being kept in culture. Cell culture conditions such as culture medium is per se selective for different subpopulations of cells, and therefore, we compared three different culture media for amnion in regard to morphology (visualized with HTX/eosin) and viability of the cells (Ki67 for proliferation and caspase 3 for apoptosis). The HTX/eosin staining showed no morphological differences in the amnion-residing cells despite different culture media, and there were no significant dissimilarities in cell viability at any time points. When the amnion was in direct contact with the culture surface, cells (ADC) migrated from the amnion and multiplied. When cultured in DMEM-F12, the ADC had a nonfibroblast and nonepithelial morphologies whereas when cultured in MEM*α* and DMEM-EC, the ADC had a fibroblast-like phenotype. We next investigated the ADC in a flow cytometric assay for surface marker expression. We chose established markers for epithelial (PCK), mesenchymal (CD73), and endothelial (CD31) cells. All ADC expressed CD73, but no PCK or CD31 expression was detected, indicating that mesenchymal and not epithelial or endothelial cells had migrated from the amnion. ADC cultured in DMEM-EC had the highest percentage of CD73-expressing cells, fibroblastic morphology and with no differences in protein expression of the amnion-residing cells between the culture medium, and DMEM-EC was used for the forthcoming analyses.

Amnion and MNC-enriched PRGF gel were combined with a matrix and thereafter analyzed. Conventional commercially available biological grafts usually consist of decellularized ECM proteins, mainly collagens, from various tissue sources such as skin (human, bovine, and porcine) and pericardium (equine and bovine) [35]. One of the most commonly used grafts is SIS (porcine) [36], and since it is currently used in the clinic, is well studied, non-cross-linked, and has a good mechanical strength, we decided to use 4-layer SIS in this study. We wanted to investigate if the properties of the amnion-residing cells would change over time when combined with SIS, and therefore, we kept the constructs in culture for up to 21 days before analysis. We stained the constructs for PCK, CD73, CD31, Ki67, and caspase 3. After 21 days of culture, the amnion remained highly viable with a low apoptotic environment as indicated by few caspase 3-positive cells, and little activation of cell proliferation, as indicated by Ki67-positive cells. Also, the cells present in the amnion when combined with SIS did not alter their phenotype, and both the epithelial and stromal cells had a stable protein expression throughout the analysis period. However, the morphological investigations showed a reduction of the epithelial cell layer integrity over time, which means that prolonged storage is not possible or require further optimization.

In the amnion-SIS constructs, the amnion-residing cells did not migrate or integrate into the SIS matrix. One explanation to this could be the manufacturing process of SIS where the tissue is harshly decellularized and sterilized, which may impact on the collagens and thereby change the protein properties and influence the biocompatibility and host integration. The processing is required for the graft to persist rapid degradation by endogenous collagenases [37, 38]. Spelzini et al. could show integration of rat mesenchymal stromal cells into SIS, but they were isolated single cells in suspension and not cells migrating from a tissue [39]. In our construct, the SIS only acts as support and will gradually degrade over time as endogenous extracellular tissue forms and replaces the SIS. By these means, cell migration into the SIS is not of major significance. In summary, these results demonstrate that in cases of delayed correction of the defect, the amnion-SIS constructs could be kept in culture for 21 days, indicating a potential for short-term storage of the construct.

The PRGF-SIS constructs were characterized, which showed that the MNC in the PRGF gel, with or without SIS, maintained a robust protein expression of both CD45 and CD34 over time. No increased proliferation (Ki67) was detected but a small increase in caspase 3 expression was observed. The increased number of apoptotic cells might be explained by a loss of PRGF-derived growth factors and cytokines at medium changes through the degradation process. The gel in the PRGF-SIS constructs slowly degraded and was completely dissolved at day 14. PRGF releases growth factors and cytokines; promotes cell recruitment, tissue regeneration, angiogenesis, and reperfusion of damaged tissue; reduces apoptosis [33, 40–42], properties that promote tissue formation, and wound healing; and reduces the risk for bacterial colonialization [43]. For these reasons, degradation of the gel within 14 days may not be a concern in a clinical setting.

When preparing the PRGF gel, which is induced clotting, we allowed a small fraction of RBC to be included, following an established protocol [33]. Even though RBC may be toxic to the cells, other studies have shown that the maximum recovery of platelets and leucocytes is when a small portion of the RBC fraction is included in platelet-enriched blood products [34].

Platelet-enriched derivatives from blood have previously been characterized in regard to platelet concentration and growth factor and cytokine content [43, 44]. In our study, the PRGF was also enriched with MNC, and similarly, the cellular content of UCB is well known [45]. Therefore, we judged it unnecessary to include such characterizations.

## 5. Conclusions

This study demonstrates that amnion and MNC-enriched PRGF gel, after minimal handling, are promising candidates as noninvasive sources for autologous grafts in correction of congenital soft tissue defects. Further studies are needed to evaluate the full potential of these constructs.

## Figures and Tables

**Table 1:** Components of the two studied constructs.

Amnion-SIS constructs	PRGF-SIS constructs
Amnion from term placenta collected from healthy donors undergoing elective caesarean section	Term umbilical cord blood collected from healthy donors was used for isolation of the following:
	(i) Plasma rich in growth factors (PRGF)
	(ii) Mononuclear cells (MNC)

Commercially available 4-layer small intestine submucosa (SIS) that consists of cell-free porcine extracellular matrix, mainly collagen	Commercially available 4-layer small intestine submucosa (SIS) that consists of cell-free porcine extracellular matrix, mainly collagen

The components were combined by suturing the amnion onto the SIS.	The components were combined by the following:
	(1) Mixing MNC with PRGF
	(2) Casting the MNC-PRGF gel onto SIS

## Supplementary Materials

Supplementary materials Supplementary figure 1. Protein expression of amnion-residing cells. Amnion cultured in DMEM-EC was analyzed for protein expression at day 0 to 21. All cells expressed CD73 and the epithelial cells expressed PCK at all time points. There was a low expression of Ki67 and caspase 3 up to day 14, and at day 21 no Ki67 expression was detected but a slightly increased caspase 3 expression. Positive cells stain brown. Magnification 20x (HTX/eosin), 40x (CD73 and PCK) and 60x (Ki67 and caspase 3). Supplementary figure 2. Morphology and protein expression of MNC in PRGF gel. MNC-enriched PRGF gel was kept in culture for up to 14 days and thereafter analyzed. The HTX/eosin staining showed decreasing number of cells and also a degradation of the gel to a total absence at day 14. The cells expressed CD45 and few CD34 positive cells were detected throughout the time points. Cells positive for Ki67 were detected, but with decreasing number over time. The caspase 3 expression was also low but increased over time. Positive cells stain brown. Magnification 60x.
