# Supplementary material for: Soft Tissue Repair with Easy-Accessible Autologous Newborn Placenta or Umbilical Cord Blood in Severe Malformations: A Primary Evaluation
Source: Stem Cells Int. 2017 Dec 17;2017:1626741. doi: 10.1155/2017/1626741 (PMC5748284; doi:10.1155/2017/1626741)
Supplement: Supplementary materials — Supplementary figure 1. Protein expression of amnion-residing cells. Amnion cultured in DMEM-EC was analyzed for protein expression at day 0 to 21. All cells expressed CD73 and the epithelial cells expressed PCK at all time points. There was a low expression of Ki67 and caspase 3 up to day 14, and at day 21 no Ki67 expression was detected but a slightly increased caspase 3 expression. Positive cells stain brown. Magnification 20x (HTX/eosin), 40x (CD73 and PCK) and 60x (Ki67 and caspase 3). Supplementary figure 2. Morphology and protein expression of MNC in PRGF gel. MNC-enriched PRGF gel was kept in culture for up to 14 days and thereafter analyzed. The HTX/eosin staining showed decreasing number of cells and also a degradation of the gel to a total absence at day 14. The cells expressed CD45 and few CD34 positive cells were detected throughout the time points. Cells positive for Ki67 were detected, but with decreasing number over time. The caspase 3 expression was also low but increased over time. Positive cells stain brown. Magnification 60x. [file 1626741.f1.docx]

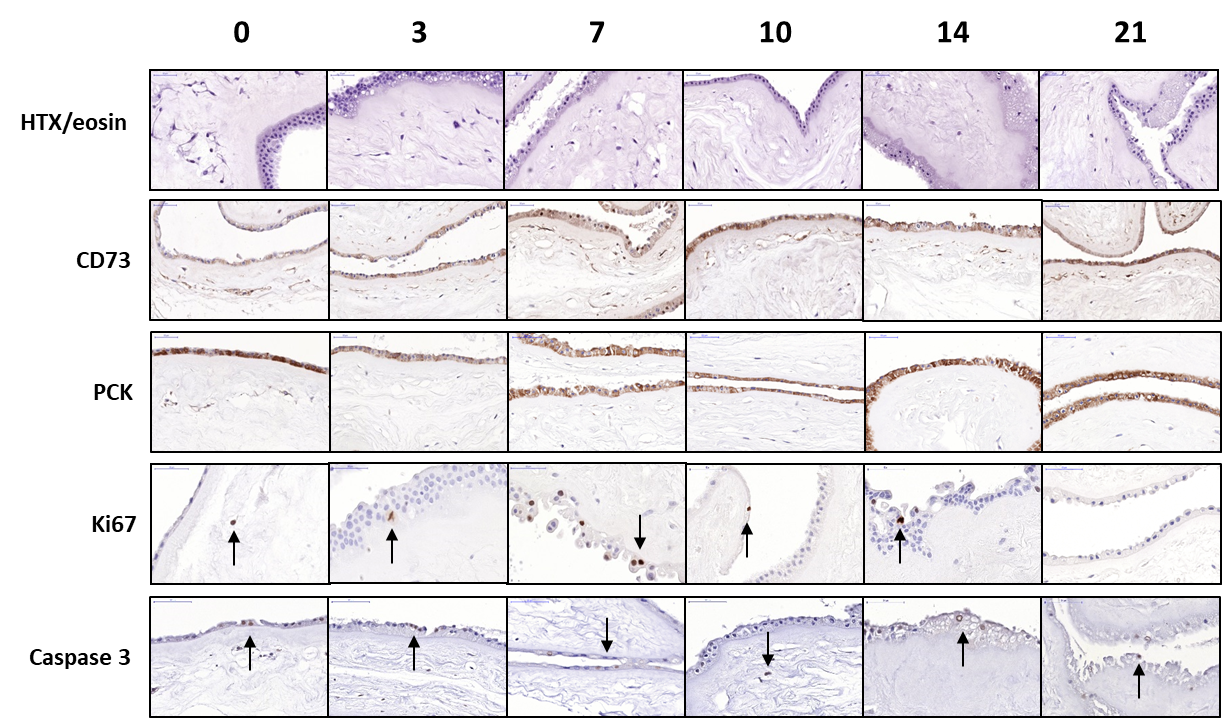


**Supplementary figure 1. Protein expression of amnion-residing cells.**

Amnion cultured in DMEM-EC was analyzed for protein expression at day 0 to 21. All cells expressed CD73 and the epithelial cells expressed PCK at all time points. There was a low expression of Ki67 and caspase 3 up to day 14, and at day 21 no Ki67 expression was detected but a slightly increased caspase 3 expression. Positive cells stain brown. Magnification 20x (HTX/eosin), 40x (CD73 and PCK) and 60x (Ki67 and caspase 3).


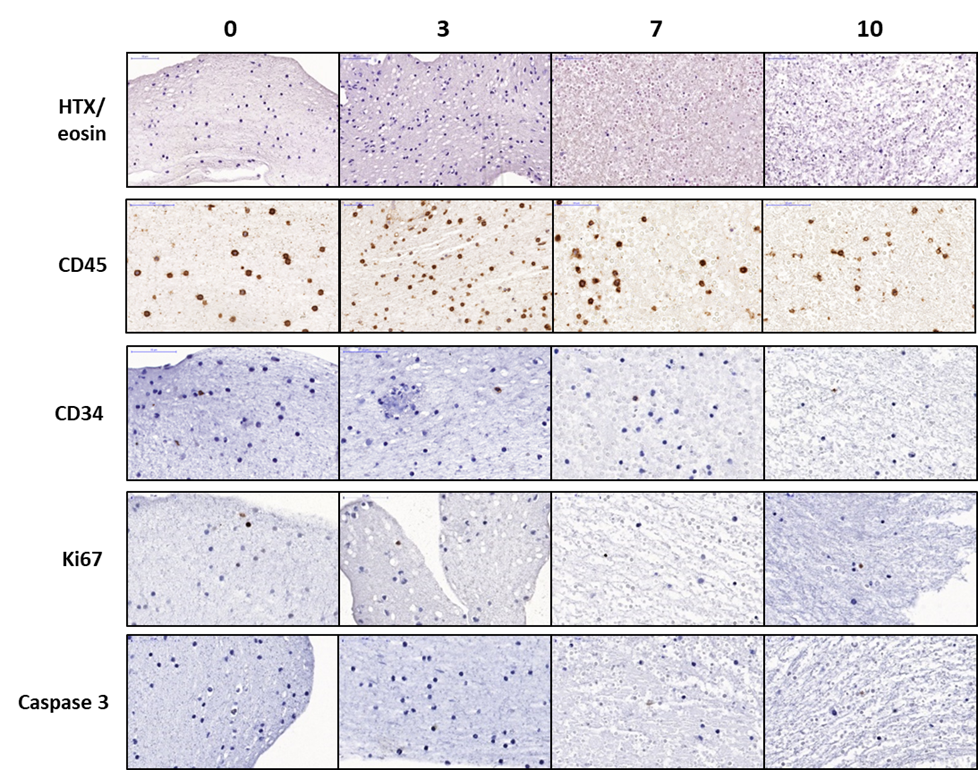


**Supplementary figure 2. Morphology and protein expression of MNC in PRGF gel.**

MNC-enriched PRGF gel was kept in culture for up to 14 days and thereafter analyzed. The HTX/eosin staining showed decreasing number of cells and also a degradation of the gel to a total absence at day 14. The cells expressed CD45 and few CD34 positive cells were detected throughout the time points. Cells positive for Ki67 were detected, but with decreasing number over time. The caspase 3 expression was also low but increased over time. Positive cells stain brown. Magnification 60x.
